# Interactive implementations of thermodynamics-based RNA structure and RNA–RNA interaction prediction approaches for example-driven teaching

**DOI:** 10.1371/journal.pcbi.1006341

**Published:** 2018-08-30

**Authors:** Martin Raden, Mostafa Mahmoud Mohamed, Syed Mohsin Ali, Rolf Backofen

**Affiliations:** 1 Chair of Forest Growth and Dendroecology, University of Freiburg, Freiburg, Germany; 2 Bioinformatics Group, Department of Computer Science, University of Freiburg, Freiburg, Germany; 3 Center for Biological Signaling Studies, University of Freiburg, Freiburg, Germany; 4 Center for Biological Systems Analysis, University of Freiburg, Freiburg, Germany; Genome Quebec, CANADA

## Abstract

The investigation of RNA-based regulation of cellular processes is becoming an increasingly important part of biological or medical research. For the analysis of this type of data, RNA-related prediction tools are integrated into many pipelines and workflows. In order to correctly apply and tune these programs, the user has to have a precise understanding of their limitations and concepts. Within this manuscript, we provide the mathematical foundations and extract the algorithmic ideas that are core to state-of-the-art RNA structure and RNA–RNA interaction prediction algorithms. To allow the reader to change and adapt the algorithms or to play with different inputs, we provide an open-source web interface to JavaScript implementations and visualizations of each algorithm. The conceptual, teaching-focused presentation enables a high-level survey of the approaches, while providing sufficient details for understanding important concepts. This is boosted by the simple generation and study of examples using the web interface available at http://rna.informatik.uni-freiburg.de/Teaching/. In combination, we provide a valuable resource for teaching, learning, and understanding the discussed prediction tools and thus enable a more informed analysis of RNA-related effects.

## Author summary

RNA molecules are central players in many cellular processes. Thus, the analysis of RNA-based regulation has provided valuable insights and is often pivotal to biological and medical research. In order to correctly select appropriate algorithms and apply available RNA structure and RNA–RNA interaction prediction software, it is crucial to have a good understanding of their limitations and concepts. Such an overview is hard to achieve by end users, since most state-of-the-art tools are introduced on expert level and are not discussed in text books. Within this manuscript, we provide the mathematical means and extract the algorithmic concepts that are core to state-of-the-art RNA structure and RNA–RNA interaction prediction algorithms. The conceptual, teaching-focused presentation enables a detailed understanding of the approaches using a simplified model for didactic purposes. We support this process by providing clear examples using the web interface of our algorithm implementation. In summary, we have compiled material and web applications for teaching—and the self-study of—several state-of-the-art algorithms commonly used to investigate the role of RNA in regulatory processes.

This is a *PLOS Computational Biology* Education paper.

## Background

Bioinformatics analyses have become indispensable to biological research. While platforms like Galaxy enable the setup of tool pipelines without expert knowledge [1, 2], one requires a general understanding of underlying concepts and algorithms to be able to successfully apply and adapt these pipelines to biological data [3, 4]. Thus, bioinformatics is taught n both computer science and biology studies.

It has been established that, when teaching mathematics, a combination of reflective example study and problem solving by hand fosters learning. This learning effect is heightened when done iteratively with increasing difficulty [5]. Thus, diverse examples covering different aspects of the topic have to be provided to guide the learning process. This is even more important in an e-learning or self-study context, in which the study of examples that show different aspects of a problem might compensate for the missing interaction with a teacher [6, 7].

Here, we focus on RNA-related bioinformatics and especially on approaches for RNA structure and RNA–RNA interaction prediction. Both are essential when investigating the vast amount of regulatory RNA that is common to all kingdoms of life [8, 9]. The function of many RNA species is guided by their structure that is defined by the formation of intramolecular base pairs. For instance, prokaryotic small RNAs show evolutionary conserved unstructured regions that regulate the expression of their target mRNAs via intermolecular base pairing [10, 11]. Thus, the prediction of both functional intramolecular structures of RNAs as well as their intermolecular (RNA–RNA) interaction potentials are central bioinformatics tasks.

Most computational methods for RNA structure or RNA–RNA interaction prediction are based on thermodynamic models and provide an efficient computation, since Richard Bellman's principle of optimality [12] can be applied. This means that optimal solutions of a problem can be composed of optimal solutions of (independent) subproblems. This is used by dynamic programming approaches that decompose a problem into smaller problems and tabularize partial solutions. Robert Giegerich and colleagues developed a rigorous framework, namely Algebraic Dynamic Programming (ADP) [13, 14], to systematically study and develop dynamic programming approaches in a computer science context. In addition, they provided an online platform to study ADP programs for various problems also covering RNA related topics [15]. The central idea of ADP is to separate the strategy of how a problem is decomposed into subproblems from the evaluation strategy, i.e., the objective of the optimization. We use the counting of structure alternatives for a given RNA to illustrate how dynamic programming can be applied to prediction problems. In particular, we introduce the decomposition strategy for (nested) RNA structure models.

The teaching of dynamic programming approaches is typically split into a theoretical introduction by the lecturer showing individual examples and a subsequent manual application by students in which the methods are implemented or applied to solve small-scale problems for exercise. This leads often to a very small set of examples discussed due to the high amount of work needed for manual application and the limited gain of knowledge by iterated usage of once-understood solution strategies. To increase the number of examples, e.g., to focus on different aspects of an individual method or to compare different approaches, either partial solutions have to be provided or implementations made available. Besides single instances like the Nussinov algorithm, most state-of-the-art methods and their underlying algorithmic ideas are not covered by textbooks, e.g., [16–18]. Resorting to the original literature for teaching these algorithms, however, is complicated, as most approaches are introduced for very sophisticated energy models. While these advanced energy models are required for a successful application of these tools in real-world scenarios, they often mask the basic and transferable algorithmic ideas for the nonexpert reader since they require a high level of background knowledge.

We approach the aforementioned problems in two ways. First, we have stripped the model-specific energy details from the state-of-the-art methods for RNA structure prediction and RNA–RNA interaction prediction and present their underlying (or basic) algorithmic ideas. For that purpose, we use the most simple energy model available. State-of-the-art energy models take the structural context of base pairs into account. To this end, RNA structures are decomposed into loops (i.e., a region that is enclosed by one or more base pairs) to calculate their overall energy. However, the algorithmic principles are essentially the same when using an energy model that considers base pairs without their structural context as basic units. Since all methods are presented using the same mathematical nomenclature, relationships and differences are easy to understand. Second, we provide a web interface that provides interactive implementations of all algorithms discussed with extensive visualizations. This interface (i) helps to understand and follow the algorithms, (ii) eases the generation of interesting examples for different aspects to teach, and (iii) provides master solutions for comparison with your own calculations or implementations. Each section closes with a list of advanced questions that exemplify what can be studied and answered using the provided web interfaces available at http://rna.informatik.uni-freiburg.de/Teaching/.

RNA structure prediction topics covered within this manuscript are the formalization of RNA secondary structures and simplified energy models, computation of the number of structures with regards to the given model [19, 20], identification of the minimum free energy structure [21, 22], computation of partition functions [23], probability calculation for single base pairs and unpaired regions [23, 24], and identification of the maximum expected accuracy structure [25, 26].

RNA–RNA interaction prediction approaches are grouped according to their algorithmic idea, as in [27], into hybrid-only interaction prediction [28–30], concatenation-based/cofolding interaction prediction [31, 32], and accessibility-based interaction prediction [24, 33, 34].

## Results and discussion

In the following, we will briefly introduce the available algorithms and their respective applications to life science. Most algorithms are dynamic programming approaches. Thus, we also provide the corresponding recursions for the simplified RNA structure model, which we introduce first.

### RNA

Ribonucleic acid (RNA) is a linear molecule built from nucleotides. The ribose sugars of the nucleotides are bound via interlinking phosphate groups. Furthermore, each sugar is connected to a nitrogenous base, typically one of adenine (*A*), guanine (*G*), cytosine (*C*), or uracil (*U*). The bases can form hydrogen bonds between two (nonconsecutive) nucleotides, which is then called a base pair. Although other forms are possible, the typically considered base pairs are *G*−*C*, *A*−*U*, and *G*−*U* in both orientations. Pairing between nucleotides of the same molecule (intramolecular) defines its three-dimensional structure. In order to fulfill a certain regulatory function, typically a stable structure is needed. Thermodynamic analyses have identified base (pair) stacking as the major stabilizing force within RNA structures [35], and according energy estimates have been identified experimentally, e.g., refer to [36]. The functional structure of an RNA can regulate, e.g., other RNA molecules by direct (intermolecular) base pairing, i.e., forming base pairs between two RNAs, called RNA–RNA interactions. While the probability of an initial contact is dependent on many factors, such as concentration or location, the subsequent formation of a stable RNA–RNA interaction is assumed to follow the same thermodynamic principles as single structure formation. Thus, most ideas and parameters from RNA structure prediction are transfered to RNA–RNA interaction prediction approaches. It is important to note that thermodynamics-based approaches are again models that do not consider all factors that influence structure/interaction formation, e.g., already bound molecules, specific solution conditions, or kinetics of structure formation. Nevertheless, they typically allow for accurate predictions for the majority of RNA molecules [37].

### RNA secondary structures

In the following, we provide the mathematical framework needed to define and solve RNA-related problems. The primary structure of an RNA molecule can be described by its sequence of bases. That is, an RNA molecule of length *n* is defined by its sequence *S*∈{*A*,*C*,*G*,*U*}^*n*^ of respective International Union of Pure and Applied Chemistry (IUPAC) single-letter codes [38].

The secondary structure *P* of an RNA *S* is defined as a set of (ordered) base pairs, i.e., *P*⊂[1,*n*]×[1,*n*] with (*i*,*j*)∈*P*→*i*<*j*. Typically, it is assumed that each nucleotide can pair with at most one other nucleotide, i.e., ∀(*i*,*j*) ≠ (*p*,*q*)∈*P*:{*i*,*j*}∩{*p*,*q*} = ∅, and that only the introduced Watson–Crick or *G*−*U* base pairs are allowed, i.e., ∀(*i*,*j*)∈*P*:{*S*_*i*_,*S*_*j*_}∈{{*A*,*U*},{*C*,*G*},{*G*,*U*}} extraneous to order. Such base pairs are said to be complementary. Furthermore, to restrict computational complexity of prediction algorithms, structures are constrained to be noncrossing (nested), i.e., ∄(*i*,*j*),(*p*,*q*)∈*P*:*i*<*p*<*j*<*q*. Using noncrossing structures generally allow a good estimate of the overall structure stability. However, it is important to note that crossing base pairs do exist, albeit not as abundant as noncrossing base pairs, and contribute to the final stability of the three-dimensional shape. It is typically assumed that first noncrossing structural elements are formed that subsequently are linked via few crossing base pairs [39]. Thus, the majority of the structure can be modeled/predicted via nested base pairing, which strongly reduces the computational complexity. Finally, it is commonly enforced that pairing bases have a minimal sequence distance of *l*, also called minimal loop length, to incorporate steric constraints of structure formation. In the following, we will denote with P the set of all possible structures (also referred to as structural ensemble or structure space) that can be formed by a given sequence *S*. It has been shown that the size of the structure space P grows exponentially with sequence length *n*. For a minimal loop length *l* of 3, the growth is about 2.3^*n*^ [40].

Nested secondary structures can be visualized as outerplanar graphs in which nucleotides are represented by nodes, and edges represent base pairs or sequential backbone connections. Furthermore, dot-bracket strings can be used that encode for each position *i* whether it is unpaired “.”, it is the smaller index (opening) of a base pair “(,” or the larger (closing) index “)”.

As motivated by Ruth Nussinov and coworkers [21], we relate the stability of an RNA structure directly with its number of base pairs. Since some algorithms require explicit energy contributions of individual base pairs (e.g., McCaskill's algorithm to compute base pair probabilities), we set the energy of any base pair *E*_*bp*_ to −1 for simplification purposes. Thus, the energy of a structure is given by *E*(*P*) = |*P*|∙*E*_*bp*_. Note, this is in stark contrast to state-of-the-art RNA structure prediction approaches (e.g., using Zuker's algorithm [22]), which typically apply a Nearest Neighbor energy model [41, 42] and experimentally derived energy contributions [36]. Furthermore, all algorithms for RNA–RNA interaction prediction ignore concentration dependence and other factors influencing the duplex formation, which is typically modeled within the Nearest Neighbor model by an “initiation” energy term [24, 33, 34]. Nevertheless, the use of the simplified base pair-focused model enables a much clearer presentation of the algorithms, which is better suited (and sufficient) to understanding their ideas and mechanisms. The transfer from the simple base pair maximization to the advanced energy models, as done by Michael Zuker and Patrick Stiegler [22], is generic and can be applied to all problems discussed within this manuscript. References to extended versions and implementations are provided for each approach.

### Counting structures via dynamic programming

**Fig 1:**
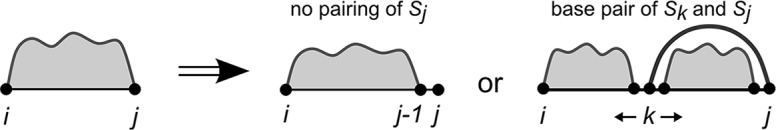
Secondary structure decomposition by Waterman and Smith (1978). The figure illustrates for a given subsequence *S*_*i*..*j*_ a unique nested secondary structure decomposition based on the distinction of all possible pairing states of the last nucleotide *S*_*j*_. Note, this scheme applies to all RNA structure-related algorithms presented here.

A first task that introduces the general structure of dynamic programming approaches used for RNA structure prediction is to compute the number of structures a sequence *S* can form, i.e., |P|. Since the structure space P grows exponentially, explicit enumeration is inefficient. In order to apply dynamic programming, we first have to have a strategy of how to decompose such a problem into independent subproblems. Let us consider the subsequence *S*_*i*_..*S*_*j*_. We can easily split the problem into two independent problems by introducing a case distinction for its last position *S*_*j*_; case (1) *S*_*j*_ is not involved in any base pairing, and case (2) *S*_*j*_ is paired with some position *S*_*k*_ (*i*≤*k*<*j*). Both cases are depicted in Fig 1. The first case can be easily reduced to a smaller problem, namely to *S*_*i*_..*S*_*j*−1_, since the unpaired position *S*_*j*_ does not allow any structural alternatives. Thus, the reduced problem directly provides a count for case 1. On the contrary, each possible base pairing of *S*_*j*_ in the second case decomposes the problem into two smaller independent problems (one to the left of and one enclosed by the base pair (*k*,*j*)), since no base pair is allowed to cross (*k*,*j*) (nestedness condition, see section on RNA secondary structure). Since any structural alternative of the left subproblem can be combined with any of the enclosed ones, we have to multiply the numbers from these smaller subproblems to get the overall count for case 2.

Michael S. Waterman and Temple S. Smith applied this idea to solve the counting problem using a table *C* [19, 20]. An entry *C*_*i*,*j*_ provides the number of structures for a subsequence *S*_*i*_..*S*_*j*_. Thus, we initialize *C*_*i*,*i*_ = 1 for all positions *i*, since any subsequence of length one is confined to the unpaired structure. The recursion for longer subsequences is given by

$$C_{i,j}=C_{i,j-1}+\sum_{\begin{array}{l} i\leq k<(j-l)\\ S_{k},S_{j}\;\mathrm{compl}. \end{array}}C_{i,k-1}\cdot C_{k+1,j-1}$$ (1)

which combines the two discussed cases to consider all possible “states” of nucleotide *S*_*j*_ in valid structures. The first (*C*_*i*,*j*−1_) covers all cases where *S*_*j*_ is unpaired, and the second counts all cases where *S*_*j*_ is paired with an *S*_*k*_ within the subsequence (second case). Note, the base pair (*k*,*j*) has to respect the minimal loop length *l*. The overall number of structures is accessed by $|P|=C_{1,n}$. Given *l* and an RNA sequence, our user interface computes and depicts the filled matrix *C*.

**Example questions**

- The decomposition and counting of RNA structures was introduced for a case distinction on *S*_*j*_. Rewrite Eq 1 using a case distinction on *S*_*i*_.
- Compute the numbers of nested structures that can be formed by random RNA sequences of different lengths. Compare the exponential growth of the structure space with the approximation 2.3^*n*^ mentioned earlier.

### Optimal structure prediction

**Fig 2:**
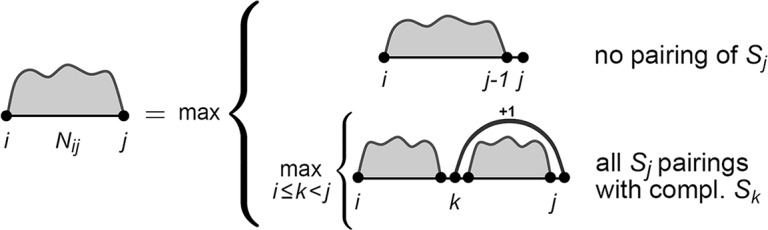
Recursion by Nussinov and coworkers (1978). The figure illustrates the recursion to compute the maximal number of base pairs that can be formed by a given sequence by distinction of all possible pairing states of the last nucleotide *S*_*j*_.

Ruth Nussinov and coworkers introduced in 1978 [21] a first algorithm that efficiently predicts a nested structure with the maximal number of base pairs for a given RNA sequence *S*, i.e., $\arg\;\max_{P\in P}(|P|)$. The corresponding recursion

$$N_{i,j}=\max\;\{\begin{array}{cc} N_{i,j-1} & S_{j}\;\mathrm{unpaired}\\ \underset{\begin{array}{l} i\leq k<(j-l)\\ S_{k},S_{j}\;\mathrm{compl}. \end{array}}{\max}\left(N_{i,k-1}+N_{k+1,j-1}+1\right) & S_{k},S_{j}\;\mathrm{pair} \end{array}$$ (2)

is strongly related to the counting approach from Eq 1. Here, an entry *N*_*i*,*j*_ stores the maximal number of base pairs that can be formed by the subsequence *S*_*i*_..*S*_*j*_. Thus, summation in Eq 1 is replaced by maximization and multiplication with summation, while the second case considers the formed base pair with “+1.” *N* is initialized with 0 and can be filled in *O*(*n*^3^) time while using *O*(*n*^2^) memory. A depiction of the recursion is given in Fig 2.

The maximal number of base pairs formed by any structure can be found in *N*_1,*n*_, and a respective optimal structure *P* can be identified via traceback starting in *N*_1,*n*_. Thus, for a given cell *N*_*i*,*j*_, the traceback discovers how the value of *N*_*i*,*j*_ was obtained. To this end, the case distinctions of the (filling) forward recursion (e.g., from Eq 2) are considered. If it holds *N*_*i*,*j*_ = *N*_*i*,*j*−1_ (first case), position *j* is found to be unpaired, and the traceback proceeds with cell *N*_*i*,*j*−1_. Otherwise, position *j* has to form a base pair with some position *i*≤*k*<*j*, which is identified in accordance to the second case of Eq 2. The base pair (*k*,*j*) is stored as part of the final structure *P* and the traceback proceeds for both subintervals represented by *N*_*i*,*k*−1_ and *N*_*k*+1,*j*−1_.

For the identification of functional structures or the study of structural alternatives, the enumeration of suboptimal structures is of interest. A generic approach was introduced by Stefan Wuchty and coworkers [43] that enables the enumeration of all structures that are in a certain range of the minimal energy. An implementation is also available in our web interface.

Our interactive user interface enables the computation of both optimal and suboptimal structures. For a user defined sequence as well as recursion and traceback parameters, the dynamic programming table is provided along with a list of (sub)optimal structures. On selection, the according traceback is highlighted within the matrix. This is complemented with a graphical representation of the structure using Forna [44].

Different recursions can be chosen to examine the effects of ambiguous recursions versus the original one. In the following, such an ambiguous variant from [17] is presented.

$$N_{i,j}=\max\;\{\begin{array}{cc} N_{i+1,j} & S_{i}\;\mathrm{unpaired}\\ N_{i,j-1} & S_{j}\;\mathrm{unpaired}\\ N_{i+1,j-1}+1 & \mathrm{if}S_{i},S_{j}\;\mathrm{compl}.\;\mathrm{and}\;i+l<j\\ \underset{i<k<(j-1)}{\max}N_{i,k}+N_{k+1,j} & \mathrm{decomposition} \end{array}$$ (3)

While this recursion also computes the same entries of *N* and thus maximal number of possible base pairs (*N*_1,*n*_), it is not using a unique decomposition of the structure, i.e., the same structural variant is considered by different recursion cases.

This causes duplicated enumeration of (sub)optimal structures when using Wuchty's traceback algorithm, which can be studied in our web server for different recursions. Furthermore, it is not possible to use variants of ambiguous recursions like Eq 3 to count structures (consider relation of Eqs 2 and 1) or to compute the partition function of the structural ensemble (as discussed next), since both requires a unique consideration of each structure.

In 1981, Michael Zuker and Patrick Stiegler introduced a dynamic programming approach that efficiently computes minimum free energy structures using a Nearest Neighbor energy model [22]. Using further restriction, the same time and space complexity compared to Nussinov's algorithm is kept. The approach with according decomposition depictions and how it relates to Nussinov's algorithm is introduced in detail, e.g., in [45]. Implementations like UNAFold [46] (formerly mfold [47]) or RNAfold [31, 37] are the current state-of-the-art tools for RNA secondary structure prediction.

**Example questions**

- Find RNA sequences that fold uniquely into (i) a single hairpin, (ii) two hairpins, and (iii) three hairpins. What guided your design?
- Find an RNA sequence that shows the ambiguity of Eq 3. What are the differences to Eq 2 that cause this ambiguity?
- Define formally what is represented by the entry *N*_1,*n*_ when using an energy minimizing variant of Eq 2 that uses *E*_*bp*_ instead of “+1.” Provide a recursion to compute this value.

### Partition function and probabilities

To estimate the probability of a given structure *P* within the structural ensemble P, statistical mechanics typically dictate a Boltzmann distribution when using minimal assumptions [48]. Thus, the probability of a structure *P* is directly related to its energy *E*(*P*) by

$$\mathrm{Pr}(P)=\frac{\exp(-E(P)/k_{B}T)}{\sum_{P^{\prime }\in P}\exp(-E(P^{\prime })/k_{B}T)}$$ (4)

given the Boltzmann factor *k*_*B*_ and the system's temperature *T*. Note, when using an energy model with units “per mole,” which is typically the case when using a Nearest Neighbor model with measured energy contributions, one has to replace *k*_*B*_ with the gas constant *R*. Note further, the structure with minimal free energy, e.g., predicted with algorithms discussed above, will always have maximal probability according to Eq 4. Thus, the most stable structure is automatically the most likely structure.

The nominator of Eq 4 is called Boltzmann weight (of structure *P*). The denominator is called canonical partition function *Z*, which is the sum of the Boltzmann weights of all structures in P. Since P grows exponentially, its exhaustive enumeration to compute *Z* is impracticable.

Nevertheless, it is possible to compute *Z* efficiently using a variant of the counting algorithm. This approach was first introduced for the Nearest Neighbor energy model by John S. McCaskill (1990) [23], and we rephrase a variant for the simplified base pair model. First, we have to note that the Boltzmann weight of a structure *P* can be computed based on the energy of its base pairs *E*_*bp*_, as follows

$$\exp(-E(P)/k_{B}T)=\exp\left(-\sum_{(i,j)\in P}E_{bp}/k_{B}T\right)=\prod_{(i,j)\in P}\exp(-E_{bp}/k_{B}T).$$ (5)

That is, the structure's weight is computed by the product of individual base pair weights. To simplify notation in the following, *q*^*bp*^ = exp(−*E*_*bp*_/*k*_*B*_*T*) refers to the Boltzmann weight of a single base pair. Given this, we can alter the counting recursion from Eq 1 to

$$Q_{i,j}=Q_{i,j-1}+\sum_{\begin{array}{l} i\leq k<(j-l)\\ S_{k},S_{j}\;\mathrm{pair} \end{array}}Q_{i,k-1}\cdot Q_{k+1,j-1}\cdot q^{bp}.$$ (6)

This directly provides the partition function *Z* =*Q*_1,*n*_ in *O*(*n*^3^) time.

For some approaches and research questions, probabilities of individual base pairs Pr^bp^(*i*,*j*) are of interest. This is the probability that a base pair (*i*,*j*) is formed by some structure, which can be calculated by summing up the probabilities of all structures containing (*i*,*j*), i.e.,

$${\mathrm{Pr}}^{\mathrm{bp}}(i,j)=\frac{\sum_{\begin{array}{l} P\in P\\ (i,j)\in P \end{array}}\exp(-E(P)/k_{B}T)}{Z}.$$ (7)

As for counting, the base pair (*i*,*j*) decomposes all structures into the enclosed and outer subsequence that are independent concerning base pairing. Thus, the partition functions of the according subsequences can be used to compute Pr^bp^(*i*,*j*) efficiently. To do so, we need an auxiliary matrix *Q*^*bp*^. Each entry $Q_{i,j}^{bp}$ holds the partition function for the subsequence *S*_*i*_..*S*_*j*_, with the side constraint that *i* and *j* form the base pair (*i*,*j*). If this is not possible due to noncomplementarity or the minimal loop constraint, the entry is 0. Given this, we can rewrite Eq 6 as follows

$$Q_{i,j}=Q_{i,j-1}+\sum_{i\leq k<(j-l)}Q_{i,k-1}\cdot Q_{k,j}^{bp}$$ (8)

$$Q_{i,j}^{bp}=\{\begin{array}{cc} Q_{i+1,j-1}\cdot q^{bp} & \mathrm{if}\;S_{i},S_{j}\;\mathrm{complementary}\\ 0 & \mathrm{otherwise} \end{array}$$ (9)

and compute the base pair probability using

$$\begin{array}{l} {\mathrm{Pr}}^{\mathrm{bp}}(i,j)=\frac{Q_{1,i-1}\cdot Q_{i,j}^{bp}\cdot Q_{j+1,n}}{Q_{1,n}}\\ +\sum_{p<i,j<q}{\mathrm{Pr}}^{\mathrm{bp}}(p,q)\cdot \frac{q^{bp}\cdot Q_{p+1,i-1}\cdot Q_{i,j}^{bp}\cdot Q_{j+1,q-1}}{Q_{p,q}^{bp}}. \end{array}$$ (10)

The first term in Eq 10 covers structures where (*i*,*j*) is an external base pair, i.e., not enclosed by any other base pair. The second term considers all structures in which (*i*,*j*) is directly enclosed by a base pair (*p*,*q*) and corrects the respective base pair probability Pr^bp^(*p*,*q*) by the probability of the structure subensemble that contains both base pairs and no “in-between spanning” base pair (*k*,*l*) with *p*<*k*<*i*<*j*<*l*<*q*. The latter probability is defined by the fraction within the second term. Note (again) that by using a simple energy model, we omit all the complex case distinctions, which allows one to concentrate on the main cases of algorithmic importance. In the full model, the first case would have been the same, whereas the second one would have been split to consider specifically each structural context a base pair can have.

In analogy to base pair probabilities, it is also possible to define and compute the unpaired probability Pr^ss^(*i*,*j*) of a subsequence *S*_*i*_..*S*_*j*_ (Eq 11), i.e., the probability of all structures that show no base pairing in the single-stranded subsequence.

$${\mathrm{Pr}}^{\mathrm{ss}}(i,j)=\frac{\sum_{P\in P_{i..j}^{\mathrm{ss}}}\exp(-E(P)/k_{B}T)}{Z}$$ (11)

$$\mathrm{with}\;P_{i..j}^{\mathrm{ss}}=\left\{P|{∄}_{(k,l)\in P}:k\in [i,j]\vee l\in [i,j]\right\}\subseteq P$$ (12)

The unpaired probability is also sometimes termed “accessibility,” as an unpaired region in an RNA is accessible for pairing to another RNA. For the computation of Pr^ss^(*i*,*j*), we only have to replace $Q_{i,j}^{bp}$ with 1 in Eq 10, since only the unpaired structure with energy zero has to be considered for *S*_*i*_..*S*_*j*_, which has a Boltzmann weight of 1.

Stephan H. Bernhart and coworkers provide in [49] details for the extension of the introduced recursions to the Nearest Neighbor model, which is also nicely detailed in [45]. Implementations are for instance available in the Vienna RNA package [37]. The authors also show how to reduce the time complexity of the probability computation from *O*(*n*^4^) to *O*(*n*^3^). To this end, they introduce another auxiliary matrix ${\hat{Q}}^{bp}$ that provides the “outer” partition function, which reflects only base pairs not enclosed by respective subsequences.

Our web implementation enables the computation of both base pair probabilities as well as unpaired probabilities. To provide insights into how the temperature and energy model influence structure and base pair probabilities, the user can alter the used temperature as well as *E*_*bp*_. Besides a visualization of the partition function tables *Q* and *Q*^*bp*^, the user is provided with a visualization of the base pair and unpaired probabilities using the established dot plot format (e.g., used also by UNAfold/mfold [46, 47] or RNAfold [37, 50]). Within this matrix-like illustration, each base pair probability is represented by a dot of proportional size, i.e., the higher the probability, the larger the dot and small probabilities are not visible. With a bit of visual practice, dot plots enable an easy identification of highly probable substructures and the study of structural alternatives.

**Example questions**

- Find an RNA sequence that folds uniquely into a single hairpin but shows an alternative hairpin with high base pair probabilities. What are the difficulties for such a design?
- What changes are observed for the partition functions when increasing the system's temperature? What is expected for lim*T*→∞?
- Where are subsequences with high unpaired probability typically located?

## Maximum expected accuracy

So far, individual structures were evaluated based on their number of base pairs or energy. This focus on single structures might hide that some substructures (base pairs or unpaired positions) are very common among highly probable structures but not found, e.g., in the most probable structure and thus are lost from the prediction. To face this problem, the expected accuracy can be used for structure evaluation [25, 26, 51].

Here, we follow Chuong B. Do and coworkers [25] and define the expected accuracy of a structure *P* by

acc(P)=∑(i,j)∈Pγ⋅2⋅Prbp(i,j)+∑k:(i,k),(k,j)∈PPru(k). (13)

It is basically the weighted sum of all base pair probabilities of the respective structure, together with unpaired probability estimates for all its positions *k* not involved in any base pair, i.e., features of the whole structural ensemble are mapped to individual structures. The position-wise unpaired probability is computed by

$${\mathrm{Pr}}^{u}(k)=1-\sum_{i<k}{\mathrm{Pr}}^{bp}(i,k)-\sum_{k<j}{\mathrm{Pr}}^{bp}(k,j)$$ (14)

from base pair probabilities, which is equivalent to Pr^ss^(*k*,*k*) from Eq 11. Base pair probabilities in Eq 13 are weighted by a factor of two to reflect that two sequence positions are covered. Furthermore, a weighting factor *γ* is introduced, which scales the importance of unpaired versus base pair probabilities.

Given this measure, we can compute the maximum expected accuracy (MEA) structure, i.e., a structure formed by the most accurate/likely base pairs rather than simply maximizing their number (or minimizing the overall energy). To calculate the MEA and an according structure, a variant of the Nussinov algorithm (Eq 2) can be applied, i.e.,

$$M_{i,j}=\max\{\begin{array}{cc} M_{i,j-1}+P_{j}^{u} & S_{j}\;\mathrm{unpaired}\\ \underset{\begin{array}{l} i\leq k<(j-l)\\ S_{k},S_{j}\;\mathrm{compl}. \end{array}}{\max}\left(M_{i,k-1}+M_{k+1,j-1}+2\gamma {\mathrm{Pr}}^{bp}(k,j)\right) & S_{k},S_{j}\;\mathrm{pair}, \end{array}$$ (15)

where unpaired positions are weighted by Pr^*u*^ (case 1) and base pairs with $2\gamma {\mathrm{Pr}}_{i,j}^{bp}$ (case 2). *M* is initialized with 0. The MEA is found in *M*_1,*n*_ while a corresponding structure can be identified via traceback. A recursion variant adapting Eq 3 can be found in [25].

Our MEA web interface computes base pair and unpaired probabilities using the recursions introduced above for the simplified energy model. Thus, the effects of temperature or base pair energy *E*_*bp*_ on MEA computations can be directly studied. As for the Nussinov algorithm, structure and traceback visualization is enabled as well as suboptimal MEA enumeration using our generic implementation of Wuchty's algorithm [43]. An alteration of the *γ* weighting factor for base pair probabilities provides insights into its importance for accurate structure prediction.

**Example questions**

- Compare the prediction results for MEA and base pair maximization (energy minimization). What do you observe and how could you explain your observations?
- What happens when altering the base pair probability weight *γ*?

### Hybridization-only interaction prediction

The fastest class of RNA–RNA interaction prediction approaches focuses only on the identification of the interaction site, i.e., only on the intermolecular base pairs, without considering the intramolecular structures of the interacting RNAs. To this end, the prefix-based decomposition scheme of global sequence alignment [52] can be adapted.

Given two RNA sequences *S*^1^ and *S*^2^ of lengths *n* and *m*, respectively, we denote with ${\overset{\leftarrow }{S}}_{j}^{2}$ the reversely indexed *S*^2^ to simplify the index notation, since RNA molecules interact in antiparallel orientation. The latter applies to both intra- and intermolecular base pairing. When considering *S*^1^ and ${\overset{\leftarrow }{S}}_{j}^{2}$, we can design a dynamic programming approach for the simplified energy model using a two-dimensional matrix *H*. An entry *H*_*i*,*j*_ will provide the maximal number of intermolecular base pairs for the prefixes $S_{1..i}^{1}$ and ${\overset{\leftarrow }{S}}_{1..j}^{2}$.

**Fig 3:**
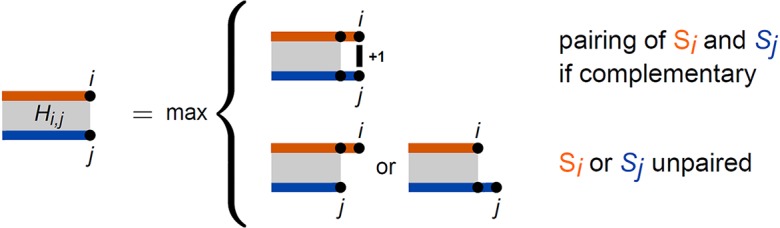
Recursion scheme to maximize intermolecular base pairs between two RNAs *S*^1^ and *S*^2^ represented in orange/blue, respectively. The optimal number for the interaction of $S_{1..i}^{1}$ and $S_{j..n}^{2}$ is identified based on a distinction whether or not the ends $S_{i}^{1}$ and $S_{j}^{2}$ might form a base pair or not.

The decomposition scheme for the recursion of Eq 16 to compute *H*_*i*,*j*_ is visualized in Fig 3.

$$H_{i,j}=\max\;\{\begin{array}{cc} H_{i-1,j-1}+1 & \mathrm{if}\;S_{i}^{1},{\overset{\leftarrow }{S}}_{j}^{2}\;\mathrm{are}\;\mathrm{complementary}\\ H_{i-1,j} & \\ H_{i,j-1} & \end{array}.$$ (16)

As already mentioned, Eq 16 is a variant of the global sequence alignment approach introduced by Saul B. Needleman and Christian D. Wunsch [52] using an adapted scoring scheme (base pair instead of match/mismatch scoring for $S_{i}^{1},{\overset{\leftarrow }{S}}_{j}^{2}$ and no gap cost). Thus, initializing all *H*_*i*,0_/*H*_0,*j*_ with 0, the entry *H*_*n*,*m*_ provides the maximal number of intermolecular base pairs that can be formed, and a traceback starting at *H*_*n*,*m*_ yields the respective interaction details. This approach enables very low runtimes (*O*(*nm*)), as observed by Brian Tjaden and coworkers, who presented in [30] a variant of Eq 16. When computing hybridization-only interactions via minimizing a more sophisticated energy model, the strategy has to be altered to follow a scheme similar to local sequence alignment as defined by Temple Smith and Michael S. Waterman [53], which is detailed in [30].

The web interface of our implementation identifies and reports all optimal interaction sites. For each, an American Standard Code for Information Interchange (ASCII) visualization of the intermolecular base pairs is provided. Note, to reduce code redundancy, we do not use an implementation of Eq 16 but use a base pair-maximization variant of Eq 19, which is discussed in the next section.

Adaptations of this approach to the Nearest Neighbor model have been discussed in [28] and, e.g., implemented in the tools TargetRNA [30], RNAhybrid [29], or RNAplex [54]. While such methods have been successfully applied for target site identification of very short RNAs, they often overestimate the length of target sites since intramolecular base pairing is ignored [33, 54]. These problems are tackled by concatenation- and accessibility-based approaches discussed next.

**Example questions**

- Provide a variant of Eq 16 that uses the original sequence *S*^2^ and according indexing, i.e., entry *H*_*i*,*j*_ provides the maximal number of intermolecular base pairs for $S_{1..i}^{1}$ and $S_{1..j}^{2}$. Think about the computation order of entries for this matrix.
- Develop a dynamic-programming recursion for hybridization-only RNA–RNA interaction prediction (base pair maximization) that restricts the lengths of unpaired subsequences enclosed by interacting base pairs. What is the runtime complexity of your recursion?

### Concatenation-based RNA–RNA interaction prediction

Among the first approaches to predict the interacting base pairs for two RNA molecules are concatenation-based or cofolding approaches [31, 32]. Here, two or more RNA sequences are concatenated into a single sequence with special interspacing linker sequences. The resulting hybrid sequence is used within an adaptation of a standard structure prediction that takes special care of the linker sequences. The linked sequences are forbidden to form base pairs, and the structural elements containing linker sequences are treated energetically as external, as discussed by Ivo L. Hofacker and colleagues [31].

The extension of standard structure prediction approaches to RNA–RNA interaction prediction directly yields the possibility to compute according probabilities of interaction sites or intermolecular base pairs [55]. A first implementation of concatenation-based prediction using the Nearest Neighbor energy model was reported for mfold [47] and later implemented in, e.g., the tools MultiRNAFold [56] and RNAcofold [55].

Our implementation extends the Nussinov recursion from Eq 2 with a special handling for linker sequence characters “X.” Base pairs (case 2) are not allowed to involve a linker position. No special energy treatment is necessary for the simplified energy model since we treat intra- and intermolecular base pairs equally and without considering their context. The input is restricted to two RNA sequences that are concatenated by a linker of length *l*+1 (where *l* is the minimal loop size) to ensure the presence of a linker and that the concatenated sequence ends can form a base pair.

Our interactive cofolding web interface lists (sub)optimal hybridization structures using our generic suboptimal traceback implementation. Within the reported dot-bracket strings, intramolecular base pairs are encoded using parentheses “(),” intermolecular base pairs (spanning the linker) are represented by brackets “[],” and the linker itself is depicted by linker characters “X.” For each hybridization structure, a traceback is visualized on selection along with a Forna 2D structure graph visualization. Furthermore, an ASCII visualization of only the intermolecular base pairs is provided.

**Fig 4:**
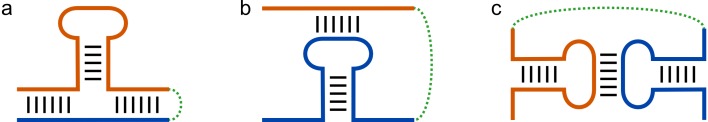
RNA–RNA interaction examples. (a) an interaction pattern that can be predicted by cofolding algorithms but not using standard accessibility-based methods, and a (b) kissing stem–loop or (c) kissing hairpin interaction pattern, both cannot be predicted by cofolding but using accessibility-based approaches. The RNA molecules are depicted in orange and blue, while the linker is indicated in dotted green. Base pairs are illustrated in black.

Concatentation-based approaches do incorporate the competition of intra- and intermolecular base pairing, which is a central weakness of hybridization-only prediction algorithms. Still, not all important interaction patterns can be predicted using cofolding approaches since the hybrid structure has to be nested. For instance, common kissing stem–loop or kissing–hairpin interactions cannot be predicted because they form a crossing structure in the concatenated model (see Fig 4). To predict such patterns, accessibility-based approaches, discussed next, can be applied.

**Example questions**

- Find RNA sequence pairs that show (i) only or (ii) no intermolecular base pairs within optimal structures. Study the suboptimals of the latter. Is it possible to find sequence pairs that do not prefer (among optimals) but still enable intermolecular base pairs (within suboptimals) using this model?
- Find example sequences for the interaction patterns from Fig 4. For Fig 4B, find a sequence that can theoretically form all base pairs of the given pattern, but no suboptimal prediction contains all pairs at the same time. Think of other patterns that cannot be predicted by concatenation-based approaches and try to find corresponding sequences.
- Find an RNA sequence pair that shows more intermolecular base pairs within optimal hybrid structures using a hybrid-only approach compared to a concatenation-based prediction. What is key to finding such sequences?

### Accessibility-based interaction prediction

The previously introduced concatenation-based approaches directly reflect the competition of intra- and intermolecular base pairing by optimizing both at the same time. Nevertheless, they are neglecting that the intramolecular structure is established before an intermolecular interaction is formed. That is, intramolecular base pairs (might) have to be opened/broken such that intermolecular base pairs can form a stable interaction. To be favorable, the interaction energy must outweigh the energy needed to make the subsequences accessible. This two-step process is modeled by accessibility-based interaction prediction approaches.

**Fig 5:**
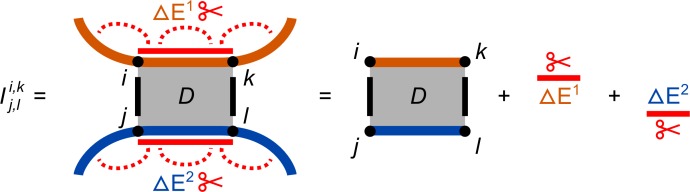
Depiction how accessibility-based approaches score an interaction of two RNAs *S*^1^ and *S*^2^ in orange and blue, respectively. The final interaction energy $I_{j,l}^{i,k}$ is only defined for subsequence combinations enclosed by two intermolecular base pairs (*i*,*j*),(*k*,*l*), marked in black. It is composed of the duplex contribution $D_{j,l}^{i,k}$ (via intermolecular base pairs), shown in grey and the energy needed to break the intramolecular base pairing of each subsequence, i.e., Δ*E*^1^+Δ*E*^2^, depicted in red.

The following formula, depicted in Fig 5, is used to compute the final interaction energy values $I_{j,l}^{i,k}$ that incorporate both the hybridization/duplex energy *D* as well as the penalties Δ*E*^1^,Δ*E*^2^ for inaccessible sites of the RNAs *S*^1^,*S*^2^, respectively.

$$I_{j,l}^{i,k}=D_{j,l}^{i,k}+\Delta E_{i..k}^{1}+\Delta E_{j..l}^{2}.$$ (17)

Note, $\Delta E_{j..l}^{2}$ is computed for the reversely indexed sequence ${\overset{\leftarrow }{S}}^{2}$ to ease the notation. This reversal has to be taken into account for hybridization energy computations, since Nearest Neighbor models have to incorporate the chemical 5′- to 3′-end orientation of RNAs. The entry of *I* with minimal energy is used to traceback the interaction details of the optimal interaction. Only entries in *I* with an energy lower than zero mark favorable interactions since here, the duplex energy *D* outweighs the Δ*E* penalties to make the respective subsequences accessible.

The energy penalties Δ*E*_*i*..*j*_ resemble the free energy needed to make the interaction site *S*_*i*_..*S*_*j*_ accessible, i.e., to unfold the site's intramolecular base pairs [24, 33]. To reflect the structural flexibility of RNAs, the terms are based on the structure ensembles that can be formed rather than individual structures. The penalties can be computed from the energy difference of the structure ensemble with accessible site that is single stranded, $E_{i..j}^{\mathrm{ss}}$, versus the whole structure ensemble, *E*^ens^. Both energies can be computed from the respective partition functions $Z_{i..j}^{\mathrm{ss}}$ (for $P_{i..j}^{\mathrm{ss}}$ from Eq 12) and *Z* using the inverse Boltzmann weight. In the following, we show the relation of Δ*E* and the unpaired probability Pr^ss^.

$$\begin{array}{l} \Delta E_{i..j}=E_{i..j}^{\mathrm{ss}}-E^{\mathrm{ens}}\\ =-\left(RT\cdot \log(Z_{i..j}^{\mathrm{ss}})-RT\cdot \log(Z)\right)=-RT\cdot \log(Z_{i..j}^{\mathrm{ss}}/Z)\\ =-RT\cdot \log({\mathrm{Pr}}^{\mathrm{ss}}(i,j)). \end{array}$$ (18)

Note, since Pr^ss^(*i*,*j*) is ≤1, all Δ*E*_*i*..*j*_ penalties are ≥0.

To add such site-specific terms to duplex energies, we cannot simply use the prefix-based recursion from Eq 16, since *H*_*i*,*j*_ only provides the optimal value for all interaction sites with right ends $S_{i}^{1}$ and ${\overset{\leftarrow }{S}}_{j}^{2}$ and not for individual sites. Thus, for exact results, we have to relate to a subsequence-based computation that explicitly stores values for all subsequence combinations. To further simplify the recursions, we use dedicated calculations (and matrices) for the duplex energy (matrix *D*, Eq 19) and the overall interaction energy including inaccessibility penalties (matrix *I*, Eq 17). Both matrices are four-dimensional, in which an entry $D_{j,l}^{i,k}$ provides the duplex energy of the interacting sites $S_{i..k}^{1}$ and ${\overset{\leftarrow }{S}}_{j..l}^{2}$ under the assumption that the boundaries form the intermolecular base pairs (*i*,*j*) and (*k*,*l*); otherwise, the entry is set to ∞.

$$D_{j,l}^{i,k}=\min\;\{\begin{array}{cc} E_{bp} & S_{i}^{1},{\overset{\leftarrow }{S}}_{j}^{2}\;\mathrm{compl}.,i=k,j=l\\ \underset{\begin{array}{l} i<p\leq k\\ j<q\leq l \end{array}}{\min}\left(E_{bp}+D_{q,l}^{p,k}\right) & S_{i}^{1},{\overset{\leftarrow }{S}}_{j}^{2}\;\mathrm{compl}.,i<k,j<l\\ +\infty & \mathrm{otherwise} \end{array}.$$ (19)

The first case represents the initiation of a new interaction that covers only the intermolecular base pair (*i*,*j*) with according energy *E*_*bp*_. The second case extends an already-computed interaction of $S_{p..k}^{1}$, ${\overset{\leftarrow }{S}}_{q..l}^{2}$ with a new base pair (*i*,*j*), while the third case is applied if the base pair (*i*,*j*) cannot be formed or the indices violate order constraints. Note, the given recursion has an *O*(*n*^6^) time complexity due to arbitrarily large gaps in the second case. Given the typically applied thermodynamic model and statistics from known interactions, the sequential distance between neighbored intermolecular base pairs is normally restricted to a small constant <30 [24], which reduces the time complexity to *O*(*n*^4^). The space complexity can be reduced to *O*(*n*^2^), as shown in [33], by interactively computing parts of *D* for a fixed right-boundary base pair (*k*,*l*).

Our implementation provides the list of all optimal interactions and visualizes the selected interaction details using an ASCII chart. Due to the four-dimensionality of the matrices *D* and *I*, only the value $I_{j,l}^{i,k}$ for the current selection as well as the penalty tables Δ*E*^1^+Δ*E*^2^ used for computation are shown.

The interactive web interface enables a straightforward comparison of the effects and restrictions of the three different interaction prediction approaches introduced. For instance, using the simple example sequences *S*^1^ = *CCC* and *S*^2^ = *CCCGGGGGG*, the hybridization-only optimization reports (as expected) any interaction patterns of *S*^1^ with *G* nucleotides of *S*^2^. In contrast, intermolecular base pairs predicted by the cofolding approach are restricted to the 3′-end of *S*^2^ since the central *G* nucleotides are blocked by an intramolecular hairpin structure (similar to Fig 4A). Both approaches neglect that RNA *S*^2^ will first (most probably) fold into a hairpin structure (with unpaired/accessible nucleotides in the center) before both interact. Thus, it is most likely this central unpaired region of *S*^2^ where interaction formation with *S*^1^ will start. The growing interaction would have to break the already-formed intramolecular base pairs for larger interaction patterns, which is not necessarily favorable. This scenario is modeled by accessibility-based approaches, which predict interactions to be restricted to the loop region only. The resulting interaction resembles a kissing stem–loop pattern (see Fig 4B).

Note, while accessibility-based approaches are well suited to predict interaction patterns like stem–loop or kissing hairpin interactions, they are still not able to model arbitrary interaction patterns. For instance, double kissing hairpin interactions can not be modeled correctly [57].

The first accessibility-based approach RNAup for the Nearest Neighbor model was introduced by Ulrike Mückstein and colleagues [24]. While it is still among the state-of-the-art prediction tools [27], its vast runtime requirements of *O*(*n*^4^) render it inapplicable for large-scale data analyses, such as genome wide target screens. This problem was tackled by Anke Busch and coworkers with IntaRNA [33, 34], which implements a heuristic version of an accessibility-based approach that extends fast hybridization-only recursions with Δ*E* penalties. IntaRNA results in a much lower *O*(*n*^2^) time complexity [33] when using precomputed or approximate Δ*E* terms, as introduced in [58]. A detailed introduction is also given in [45]. A similar heuristic extension was recently reported for TargetRNA2 [59]. Current versions of the initially hybridization-only approach RNAplex [54] and its webserver RNApredator [60] incorporate an approximate, position-specific accessibility model to increase prediction quality [61].

**Example questions**

- Rewrite Eq 19 to directly compute the final interaction energy values from Eq 17.
- Why can interaction patterns enclosing intramolecular base pairs (see Fig 4) not be predicted by the introduced basic accessibility-based approaches?

### Implementation

All discussed algorithms and visualizations have been implemented in JavaScript. This enables client-side computation (no backend server hardware needed) as well as local download and application (from GitHub repository) for offline usage. Since all algorithms are dynamic-programming approaches, a generic inheritance hierarchy was implemented to reduce code redundancy and to simplify maintenance and extensibility. We use Knockout.js as the controller to bind input/output elements from within the HTML pages with the JavaScript data structures and computations.

## Conclusion

The understanding of RNA structure and RNA–RNA interaction prediction approaches is central to ensure correct result interpretation and an awareness of their limitations, both essential to avoid wrong conclusions. Furthermore, it ensures proper embedding in RNA-related analysis pipelines or their extension to new fields of applications.

To gain this level of understanding, the original literature is often of limited didactic value, since scientific articles are typically not meant for educational use. Thus, approaches are either represented on a very detailed expert level or sketched briefly, since the manuscript focuses on the biological results rather than algorithmic details.

Here, we provide a compact summary of the relevant theoretical background for the most common algorithmic approaches and their state-of-the-art instances currently used. Algorithms are stripped from complicating energy model details to enable an easy understanding of the underlying concepts and the resulting limitations. Furthermore, we provide web-based implementations and visualizations of all presented approaches for their ad hoc use. The latter is of importance, since example-driven (self-)study is known to significantly foster learning and understanding. To further support such self-learning efforts based on our manuscript and web service, we provide small exemplary tasks for each algorithm group that can be tackled using our web implementations.

The web service [62] is being continually extended with the implementation and visualization of additional methods. Planned implementations cover pseudoknotted (crossing) structure prediction approaches as well as comparative approaches for RNA structure and RNA–RNA interaction prediction, e.g., discussed in [57].

Eventually, we provide both a comprehensive review of current RNA thermodynamic-focused prediction approaches to spark ideas for new approaches and interactive teaching material, which will help ensure that available tools are correctly applied and interpreted.
